# Primary Laryngeal Squamous Cell Carcinoma with *DEK::AFF2* Fusion: The First Case Report

**DOI:** 10.1007/s12105-025-01793-z

**Published:** 2025-04-29

**Authors:** Alan Yan-Lun Huang, Ying-Ju Kuo, Muh-Hwa Yang, Shyh-Kuan Tai, Jen-Fan Hang

**Affiliations:** 1 Department of Pathology and Laboratory Medicine, Taipei Veterans General Hospital, No. 201, Sec. 2, Shipai Rd, Taipei City, 112201 Taiwan; 2 School of Medicine, National Yang Ming Chiao Tung University, Taipei, Taiwan; 3 Institute of Clinical Medicine, National Yang Ming Chiao Tung University, Taipei, Taiwan; 4 Cancer and Immunology Research Center, National Yang Ming Chiao Tung University, Taipei, Taiwan; 5 Department of Oncology, Taipei Veterans General Hospital, Taipei, Taiwan; 6 Department of Otolaryngology, Taipei Veterans General Hospital, No. 201, Sec. 2, Shipai Rd, Taipei City, 11217 Taiwan

**Keywords:** *DEK:AFF2* Fusion, Larynx, Non-keratinizing, Squamous cell carcinoma

## Abstract

**Background:**

*DEK::AFF2* fusion squamous cell carcinoma (SCC) is a rare and aggressive subtype of non-keratinizing SCC. Previously reported cases have predominantly involved the sinonasal tract, middle ear, and skull base. To date, only 62 cases have been described, with rare exceptions including a primary lung tumor and a recurrent tumor in the trachea.

**Methods:**

We describe the first documented case of primary laryngeal SCC harboring a *DEK::AFF2* fusion in a 64-year-old female who presented with progressive hoarseness and airway obstruction. Clinical, radiologic, histopathologic, immunohistochemical, and molecular analyses were performed to characterize the tumor.

**Results:**

Imaging and laryngoscopic evaluation revealed a transglottic mass. Histopathology demonstrated non-keratinizing SCC composed of monotonous tumor cells with an infiltrative growth pattern. Immunohistochemistry showed diffuse p40 positivity and moderate to strong nuclear AFF2 expression. The *DEK::AFF2* fusion was confirmed by fluorescence in situ hybridization and reverse transcription polymerase chain reaction. The patient underwent total laryngectomy followed by adjuvant chemoradiotherapy and remains disease-free at 12 months of follow-up.

**Conclusions:**

This case expands the known anatomical distribution of *DEK::AFF2* fusion SCC to include the larynx, suggesting that this rare entity may arise throughout the respiratory tract epithelium. Recognition of this fusion is important in the differential diagnosis of non-keratinizing SCCs across the respiratory tract.

## Introduction

The *DEK::AFF2* fusion was first discovered in a patient with unresectable squamous cell carcinoma (SCC) of the skull base by Yang et al. in 2019 [1]. Interestingly, despite a low tumor mutation burden and negative PD-L1 expression, this patient, who had lung metastases, achieved complete remission following treatment with the PD-1 inhibitor pembrolizumab. Since then, 62 cases of head and neck SCC harboring *DEK::AFF2* fusion have been reported in the English literature [1–17]. These tumors have arisen almost exclusively in the sinonasal tract and skull base, with the nasal cavity being the most commonly affected site (34/61, 55.7%), followed by the paranasal sinuses (16/61, 26.2%), middle ear (8/61, 13.1%), skull base (7/61, 11.5%), mastoid bone (7/61, 11.5%), nasopharynx (6/61, 9.8%), and orbit and/or lacrimal sac (5/61, 8.2%). Notably, involvement of multiple sites is not uncommon (15/61, 24.6%).

The diagnosis of *DEK::AFF2* carcinoma relies on a combination of immunohistochemistry (IHC) and molecular techniques. While molecular methods such as fluorescence in situ hybridization (FISH), reverse transcription-polymerase chain reaction (RT-PCR), and next-generation sequencing (NGS) are traditionally used for confirmation, their high cost, technical demands, and processing time may limit widespread application. In contrast, IHC using an anti-AFF2 C-terminus antibody has proven to be a highly sensitive and specific marker. Our previous study demonstrated that AFF2 IHC reliably identifies *DEK::AFF2* carcinoma, with tumor cells exhibiting moderate to strong nuclear staining in at least 30% of cells [4]. Notably, AFF2 IHC remains effective even when molecular testing is compromised by poor nucleic acid quality. A combined approach integrating IHC with molecular techniques provides a robust and practical diagnostic strategy, ensuring accurate identification and guiding optimal clinical management [4, 11].

To date, *DEK::AFF2* carcinoma has been considered a malignancy confined to the upper respiratory tract, with only one primary lung SCC with *DEK*::*AFF2* fusion reported in a 26-year-old female never-smoker [17]. Additionally, a 52-year-old non-smoking female, initially diagnosed with inverted papilloma in the ethmoid sinus, experienced multiple recurrences in the sinonasl tract over 24 years, ultimately presenting with a tracheal mass harboring *DEK*::*AFF2* fusion [6]. Here, we report the first documented case of primary laryngeal SCC with *DEK::AFF2* fusion, suggesting that this carcinoma may not be restricted to the sinonasal tract and skull base but could potentially arise throughout the respiratory tract epithelium.

## Methods

A panel of IHC stains, including antibodies against p40 (clone BC28; Biocare Medical, Pacheco, CA, USA), NUT (clone 52B1; Cell Signaling Technology, Danvers, MA, USA), NKX2.2 (clone 74.5A5; BD Biosciences, San Jose, CA, USA), and AFF2 (HPA003139, Sigma-Aldrich, St. Louis, MO, USA) were performed on 4-µm paraffin sections on a Ventana BenchMark ULTRA system (Ventana, Tucson, AZ, USA). Molecular confirmation, including *DEK* FISH and RT-PCR for the *DEK::AFF2* fusion, was performed as previously described [2].

## Results

### Clinical Summary

**Fig. 1:**
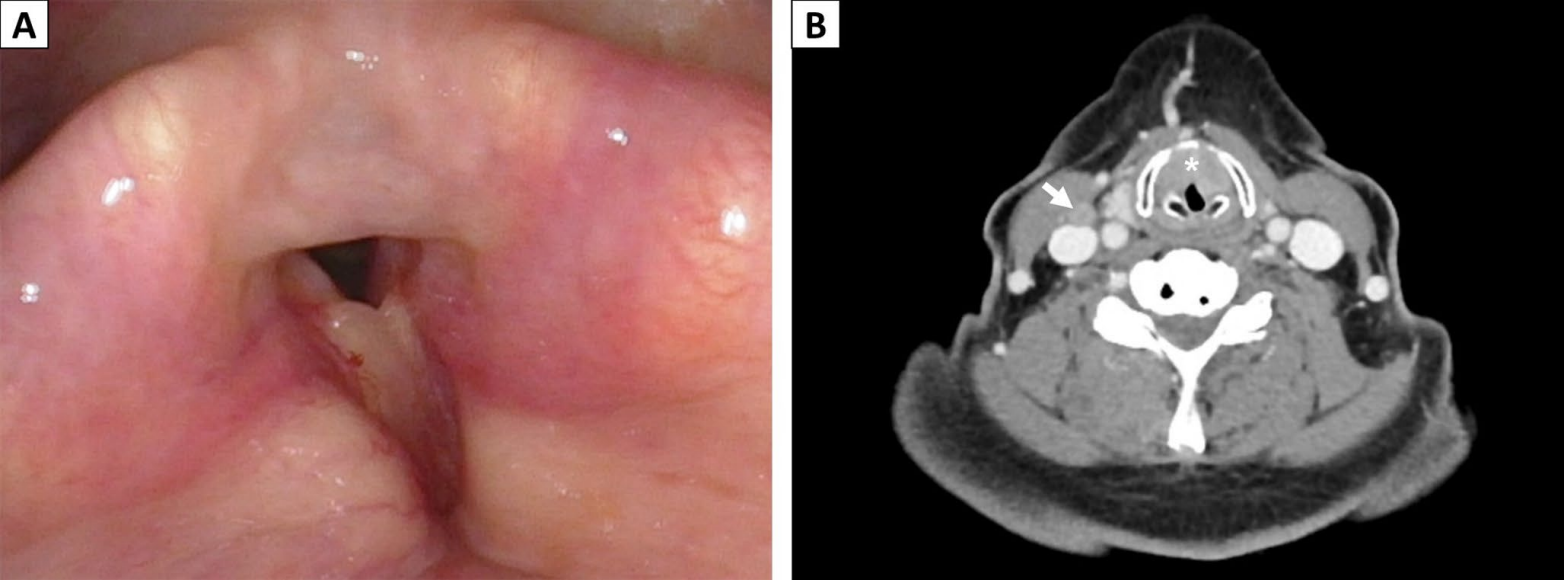
Clinical presentation. (**A**) Nasopharyngoscopy revealed adhesion of the anterior two-thirds part of the true cords. (**B**) Neck computed tomography showed increased soft tissue components involving the anterior commissure (asterisk) and the anterior part of bilateral vocal cords. One enlarged lymph node on right level III was highlighted (arrow)

The 64-year-old female presented with progressive hoarseness for one year, followed by difficulty in breathing for two months. She was a smoker, one pack/day for 26 years, and had quit since the development of these symptoms. On physical examination, biphasic inspiratory and expiratory stridor were observed. Nasopharyngoscopy revealed swelling and adhesion of bilateral anterior two-thirds of the true cords with limited abduction of the bilateral arytenoid and compromised airway (Fig. 1A). Neck computed tomography showed increased soft tissue components involving the anterior commissure and the anterior part of bilateral vocal cords and extending to the right supraglottic and bilateral subglottic regions (Fig. 1B). Transglottic laryngeal cancer was considered. In addition, multiple borderline-sized lymph nodes were identified in the bilateral level II and III areas. Due to bilateral vocal fold paralysis with impending upper airway obstruction, the patient received a tracheostomy followed by a biopsy.

### Pathological Findings

**Fig. 2:**
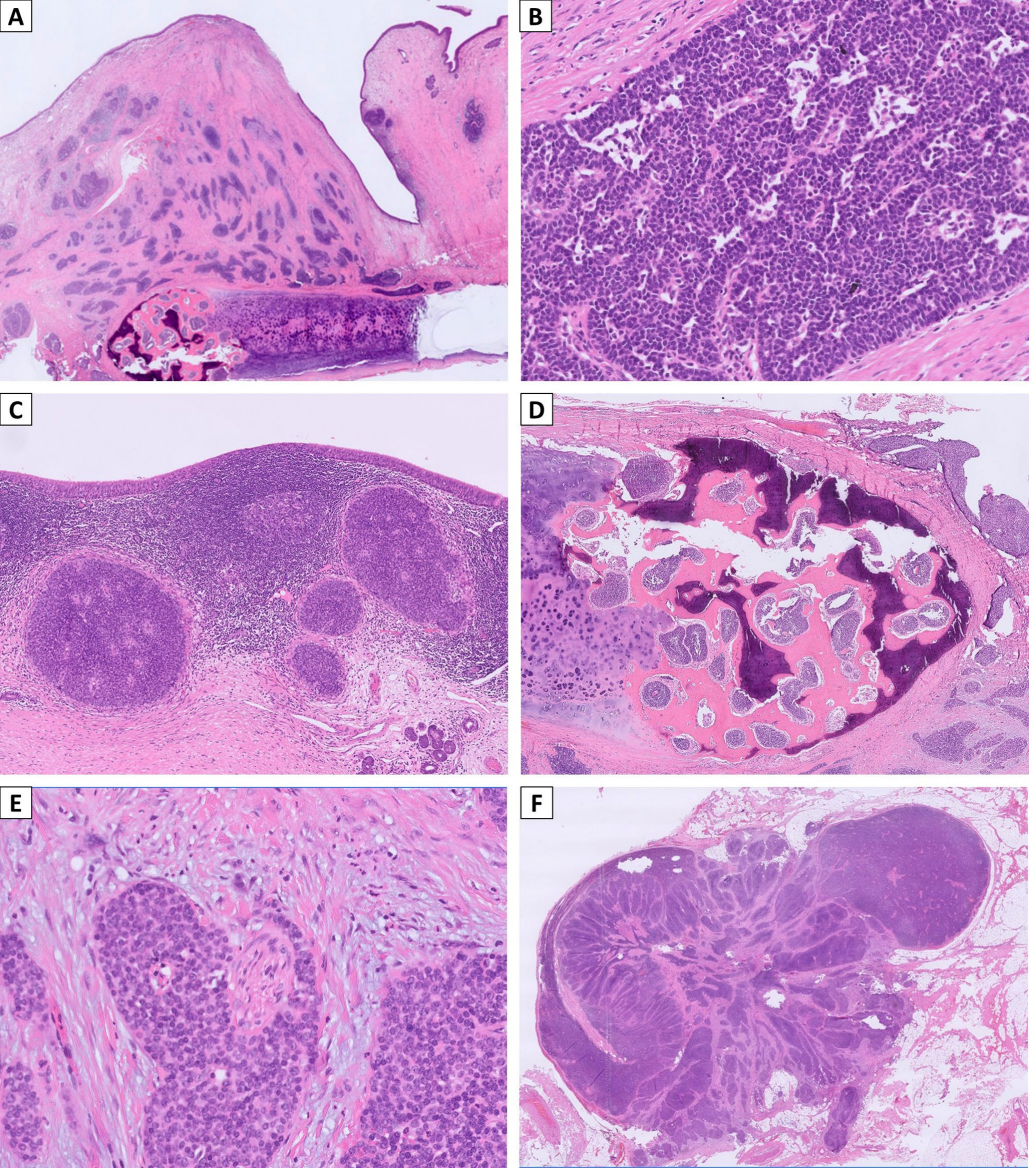
Histopathologic characteristics of laryngeal *DEK::AFF2* carcinoma. (**A**) A low-power photomicrograph showed transglottic involvement by infiltrating solid nests (caudal side toward right, false cord on the right side, true cord on the left, and thyroid cartilage at the bottom). (**B**) A high-power photomicrograph demonstrated the relatively monotonous appearance of non-keratinizing tumor cells with focal vague acantholysis and peripheral palisading. Note the absence of prominent tumor-infiltrating inflammatory cell infiltrate. (**C**) The tumor cells showed a pushing invasive pattern with an irregular contour. (**D**) The tumor involved ossified thyroid cartilage. (**E**) Perineural invasion. (**F**) Lymph node metastasis with extranodal extension. The distance of extranodal extension from lymph node capsule is 2.5 mm. (**A-F**, H&E, **A**: x10; **B**: x400; **C**: x50, **D**: x20, **E**: x200, **F**: x10)

**Fig. 3:**
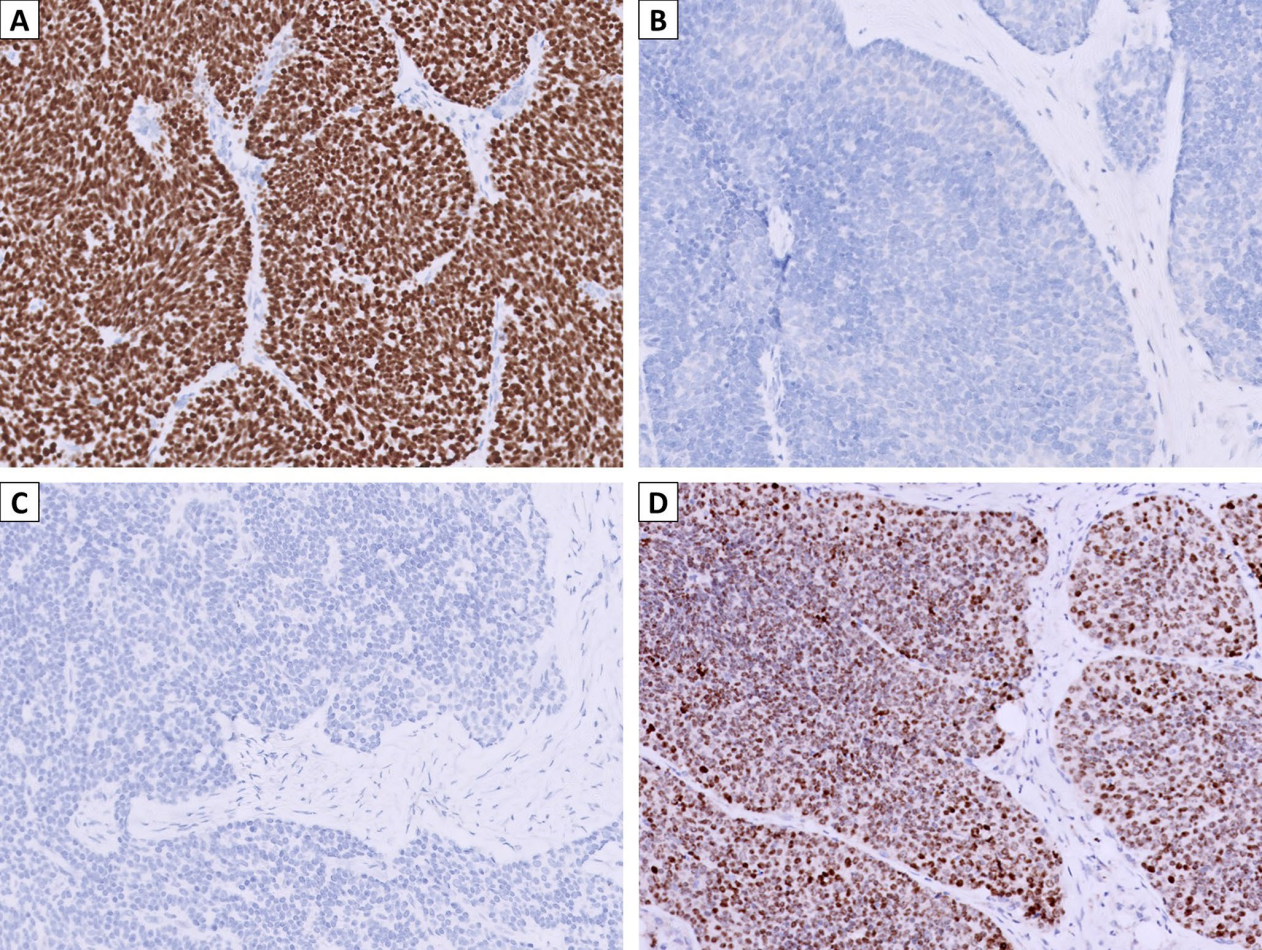
Immunohistochemical analysis of the case. (**A**) Positive p40 nuclear staining supported the squamous differentiation of tumor cells. (**B**) NUT and (**C**) NKX2.2 stains are negative. (**D**) Diffuse and moderate to strong nuclear expression of AFF2 indicated the diagnosis of *DEK::AFF2* carcinoma. (**A-D**, x200)

**Fig. 4:**
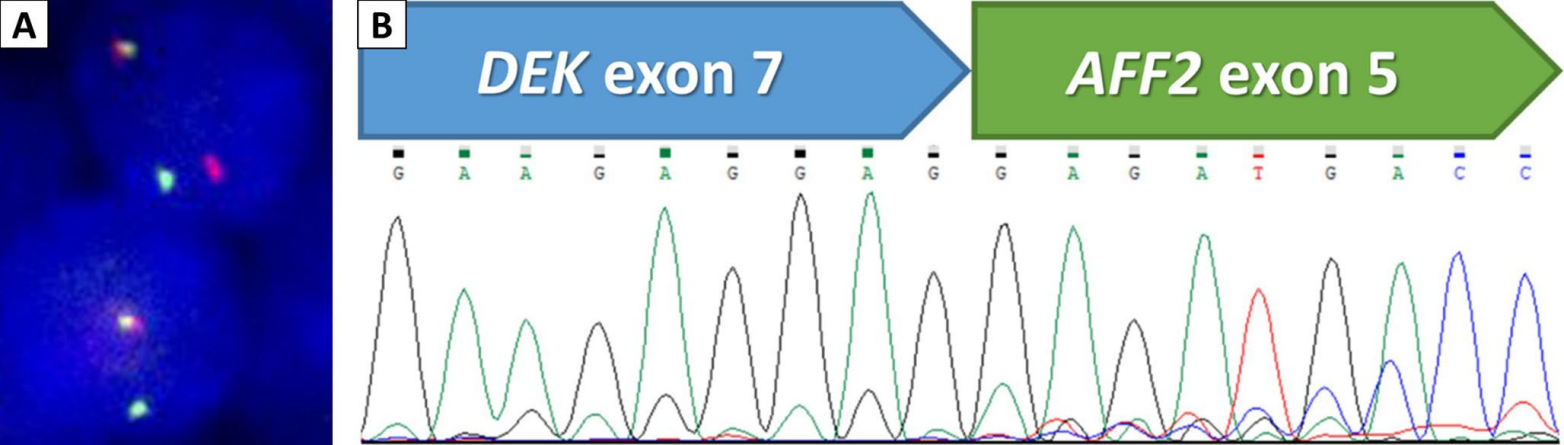
Molecular technique confirmed the *DEK::AFF2* rearrangement. (**A**) Fluorescence in situ hybridization using *DEK* break-apart probes displayed break-apart signals in tumor cells. (**B**) Reverse transcription polymerase chain reaction identified *DEKexon7::AFF2exon5* rearrangement

The pathology of the initial biopsy confirmed a non-keratinizing SCC. Therefore, a total laryngectomy with bilateral neck lymph node dissection was performed, and the final pathologic staging was pT4aN3bM0. Microscopic examination of the specimens showed transglottic involvement of non-keratinizing SCC, characterized by non-keratinizing basaloid tumor cells arranged in infiltrating solid nests (Fig. 2A). Interestingly, the tumor cells had a relatively monotonous appearance with focal vague acantholysis and peripheral palisading (Fig. 2B), distinct from the pleomorphic features of usual SCC, raising the concern of translocation-associated malignancy. No prominent neutrophilic infiltrate was seen in this case. The tumor demonstrated a pushing invasive pattern with irregular contour (Fig. 2C). It involved anterior strap muscle, thyroid cartilage, cricoid cartilage, and trachea (Fig. 2D) and showed lymphovascular and perineural invasion (Fig. 2E). Bilateral lymph node metastases were also noted, with the largest metastatic deposit of 1.2 cm and exhibiting extranodal extension with the largest dimension of 2.5 mm (Fig. 2F). A panel of immunohistochemical stains was performed. The tumor cells were diffusely positive for p40 (Fig. 3A) while negative for NUT (Fig. 3B) and NKX2.2 (Fig. 3C), which supported squamous differentiation and excluded NUT carcinoma and adamantinoma-like Ewing sarcoma. Surprisingly, diffuse and moderate to strong nuclear expression of AFF2 C-terminus was observed (Fig. 3D), suggesting the possibility of *DEK::AFF2* fusion. Subsequent *DEK* FISH demonstrated break-apart signals (Fig. 4A), and RT-PCR confirmed the presence of *DEKexon7::AFF2exon5* transcripts (Fig. 4B). Therefore, the final diagnosis was non-keratinizing SCC with *DEK::AFF2* fusion.

### Follow-Up Information

The patient made a successful recovery following surgery and underwent adjuvant concurrent chemoradiotherapy with weekly cisplatin for disease control. She is currently being closely monitored with imaging follow-up. No recurrence or significant neck lymphadenopathy has been identified on imaging studies 12 months after completing chemoradiotherapy.

## Discussion

*DEK::AFF2* carcinoma, considered a subtype of non-keratinizing SCC under the chapter of nasal, paranasal, and skull base tumors in the 2022 World Health Organization Classification of the Head and Neck Tumors, is a rare but aggressive neoplasm [18]. As noted by Skálová et al., sinonasal non-keratinizing SCC is now recognized as a molecularly defined group of distinct entities, including *DEK::AFF2* carcinoma, rather than a single descriptive diagnosis [19]. Since its initial description in 2019, 62 cases have been reported in the literature, nearly all confined to the sinonasal tract and skull base. The upper respiratory tract, specifically the nasal cavity, nasal turbinates, paranasal sinuses, and nasopharynx, has been the most affected site. Skull base and middle ear involvement have been observed in approximately 17.7% of the cases. Only two cases have been documented outside the above regions. One involved a primary lung SCC with *DEK::AFF2* fusion in a 26-year-old female [17]. Another case involved a 52-year-old non-smoking female initially diagnosed with an ethmoid inverted papilloma, with multiple recurrences as non-keratinizing SCC in the nasal cavity and maxillary sinus, ultimately manifesting as an obstructing tracheal mass that proven to be positive for *DEK::AFF2* fusion 24 years later [6].

Currently, *DEK::AFF2* carcinoma has been reported to exhibit three distinct histologic patterns: low-grade papillary Schneiderian carcinoma, high-grade non-keratinizing SCC, and adenosquamous carcinoma [20]. The most prevalent pattern, low-grade papillary Schneiderian carcinoma, is characterized by an exophytic and endophytic growth pattern with labyrinthine trabeculae, delicate papillary structures, and a monotonous basaloid to squamotransitional cytology. On the contrary, the high-grade non-keratinizing SCC pattern exhibits overtly malignant cytologic features, including enlarged vesicular nuclei, prominent nucleoli, and variable tumor necrosis, closely resembling conventional SCC. The uncommon adenosquamous carcinoma pattern is marked by a non-keratinizing squamous component accompanied by ciliated and goblet cell-containing glandular structures. While these morphologic patterns are not currently formalized in the WHO classification, they have been consistently described in the literature and may aid in the differential diagnosis, especially in small biopsies or unusual anatomical sites. Histological features established by Kuo et al. and Rooper et al. highlight key findings such as broad papillary fronds, cytologic monotony, acantholysis, and prominent neutrophilic infiltrates [2, 3]. These features, along with the absence of significant keratinization, bear a close resemblance to sinonasal papilloma [2, 6]. Moreover, rare histopathologic features such as whorl formation, clear cell transformation, microcyst development, and abrupt keratinization have been reported. Importantly, some recurrent tumors exhibit histologic progression, highlighting the need for precise diagnosis and ongoing clinical monitoring. In two reported cases initially diagnosed as sinonasal papilloma, one recurrent tumor displayed features similar to low-grade papillary urothelial carcinoma, while the other manifested as a lymph node metastasis with high-grade characteristics, including solid and cystic alterations [2]. In the current case, the morphologic features of cytologic monotony, focal subtle acantholysis, and the absence of significant keratinization particularly raise the possibility of a *DEK::AFF2* fusion. It is noteworthy that the features of neutrophilic infiltration may not present in about 20% of *DEK::AFF2* carcinoma cases [4].

The IHC plays an important role in distinguishing this carcinoma from other sinonasal tumors. *DEK::AFF2* carcinomas consistently express squamous cell markers, such as p40, CK5/6 and p63, while typically negative for synaptophysin, NUT, and p16 [3, 20, 21]. Molecular techniques, such as FISH, RT-PCR, and NGS, remain the gold standard for confirming the *DEK::AFF2* rearrangement [2, 6]. However, the limited availability and high cost of these methods restrict their routine use in many clinical settings. As an alternative, AFF2 IHC has been validated as a sensitive and specific ancillary marker for *DEK::AFF2* carcinoma, serving as a surrogate for molecular confirmation [4, 6, 14]. Diagnostic algorithms proposed by various research teams highlight the utility of combining p40, p16, AFF2, and other markers in establishing the diagnosis [5, 12].

Accurate recognition of *DEK::AFF2* carcinoma is essential to prevent misdiagnosis and ensure appropriate clinical management. Due to its frequently bland histologic appearance, it is often mistaken for benign sinonasal papillomas, potentially leading to delayed or inadequate treatment [2, 22]. According to Hart et al., although these tumors exhibit a high rate of local recurrence, patients with *DEK::AFF2* carcinoma have survival rates similar to conventional SCC, which is not correlated with morphology, mitotic activity, or the Ki-67 index [22]. Additionally, the higher rate of local recurrence may be confounded by the tumor’s deceptively benign appearance, which could lead to insufficient initial management. Therefore, an accurate diagnosis is essential to ensure optimal treatment and follow-up. In the current case, extensive local infiltration, perineural invasion, and multiple lymph node metastases with extranodal extension suggest a potentially more aggressive clinical course of *DEK::AFF2* carcinoma. Further prospective studies are needed to determine the prognostic factors of this newly recognized entity.

In conclusion, we present the first case of primary non-keratinizing SCC with *DEK::AFF2* fusion occurring in the larynx with transglottic involvement, marking an expansion of the known anatomical locations of this under-recognized entity. Previously reported cases have been largely confined to the upper respiratory tract, predominantly the sinonasal region and skull base, except of a lung primary and a tracheal recurrence. This case underscores the potential for *DEK::AFF2* carcinoma to occur throughout the entire respiratory tract, including the larynx. While earlier studies have emphasized the risk of initial misdiagnosis as sinonasal papilloma, our case highlights the possibility of *DEK::AFF2* fusion in SCCs arising in any part of the respiratory tract. Despite their sometimes paradoxically bland morphology, these tumors exhibit aggressive behavior and require a more intensive clinical approach, like other subtypes of SCC. The unique localization in the larynx emphasizes the importance of considering *DEK::AFF2* carcinoma in respiratory tract malignancies.

## Data Availability

No datasets were generated or analysed during the current study.
